# Whole-Genome Sequences of 18 Bovine Alphaherpesvirus 1 Field Isolates from Pennsylvania and Minnesota

**DOI:** 10.1128/genomeA.00294-18

**Published:** 2018-04-26

**Authors:** Shubhada K. Chothe, Aswathy Sebastian, Asha Thomas, Ruth Nissly, Maurice Byukusenge, David Wolfgang, Sunil Kumar Mor, Sagar Goyal, Istvan Albert, Bhushan Jayarao, Suresh V. Kuchipudi

**Affiliations:** a Penn State Animal Diagnostic Laboratory, Department of Veterinary and Biomedical Sciences, Pennsylvania State University, University Park, Pennsylvania, USA; b Department of Biochemistry and Molecular Biology, Pennsylvania State University, University Park, Pennsylvania, USA; c Pennsylvania Department of Agriculture, Bureau of Animal Health and Diagnostic Services, Harrisburg, Pennsylvania, USA; d Veterinary Population Medicine, University of Minnesota, St. Paul, Minnesota, USA

## Abstract

Bovine alphaherpesvirus 1 (BHV-1), a member of the Herpesviridae family, causes respiratory and reproductive tract infections in cattle. Here, we report complete genome sequences of 18 field isolates of BHV-1 from Pennsylvania and Minnesota.

## GENOME ANNOUNCEMENT

Bovine alphaherpesvirus 1 (BHV-1) is a major viral pathogen affecting cattle worldwide. BHV-1 primarily causes respiratory illness referred to as infectious bovine rhinotracheitis (IBR), along with reproductive disorders, including abortion and infertility in cattle (1). BHV-1 is a member of the family Herpesviridae. BHV-1 virions are approximately 120 to 200 nm in diameter and have an icosahedral nucleocapsid of around 100 nm in diameter that is surrounded by a protein tegument and an envelope (2). Genomes of BHV-1 strains comprise double-stranded DNA of 135 kb with 72 coding regions. The complete genome sequences of only nine BHV-1 isolates currently exist in GenBank.

We report here the whole-genome sequences of 18 BHV-1 field isolates from Pennsylvania and Minnesota. The field isolates were obtained using samples collected from local dairy farms in Pennsylvania or samples submitted to veterinary diagnostic laboratories at the University of Minnesota and Pennsylvania State University. The samples included nasal swabs, vaginal swabs, eye swabs, bronchial swabs, tissue homogenates, and stomach contents. The field isolates were subjected to viral DNA extraction using the QIAamp MinElute virus spin kit (Qiagen, Germantown, MD, USA). The total viral genomic DNA was used in a SYBR Green-based real-time PCR (RT-PCR) assay to detect the presence of BHV-1 (3). The BHV-1 RT-PCR-positive isolates were subjected to virus isolation on Madin-Darby bovine kidney (MDBK) cells. The infected cells were put through three freeze-thaw cycles, and the resulting supernatant was subjected to ultracentrifugation to obtain a concentrated virus, as previously described (2). Two hundred microliters of the concentrated virus was subjected to viral DNA extraction using the QIAamp MinElute virus spin kit.

The whole-genome sequencing was performed at the Penn State Genomics Core Facility (University Park, PA, USA). The BHV-1 DNA barcoded libraries were made using the Illumina TruSeq DNA Nano kit. An equimolar pool of the libraries was sequenced on a single 150 × 150 paired-end MiSeq run. Approximately 700,000 pairs of reads or 210 Mb of sequence was generated per isolate. Sequence quality was visualized using FastQC reports. All the field isolates were mapped to the National Veterinary Services Laboratory (NVSL) reference/challenge Cooper strain of BHV-1.1 (GenBank accession number JX898220) using the bwa-mem algorithm (4). Variants were called using freebayes (5). Low-quality variants were filtered out, and consensus sequences were obtained using the bcftools consensus tool (http://github.com/samtools/bcftools). Based on phylogenetic analysis, the isolates were all identified as belonging to the BHV1.1 genotype.

### Accession number(s).

The complete genome sequences of BHV-1 field isolates have been deposited in GenBank under the accession numbers MG407775 to MG407792.
